# Behavioral Sleep Interventions for Neurodivergent Youth: A Narrative Review

**DOI:** 10.1007/s40675-026-00379-7

**Published:** 2026-06-08

**Authors:** Amy G. Hartman, Angela Caldwell, Heather Joseph, Jessie Northrup, Amy J. Schwichtenberg, Adriane M. Soehner

**Affiliations:** 1 Department of Occupational Therapy, University of Pittsburgh, 100 Technology Dr, Suite 350, Pittsburgh, PA 15221 USA; 2 Department of Psychiatry, University of Pittsburgh, Pittsburgh, PA USA; 3 Department of Human Development and Family Science, Purdue University, West Lafayette, IN USA

**Keywords:** Sleep, Behavioral sleep intervention, Neurodevelopmental disorder, Attention deficit hyperactivity disorder, Autism, Down syndrome

## Abstract

**Purpose of this review:**

Sleep health in childhood is critical for growth and development, however, neurodivergent children (e.g., children with attention deficit hyperactivity disorder, autism, Down syndrome) experience significantly more sleep challenges compared to their peers. Recent intervention research has begun to address specific challenges that contribute to poor sleep health for neurodivergent children. This review identifies behavioral sleep interventions published in the past 5 years that are developed for neurodivergent children and examines the impact of these interventions on sleep health.

**Recent findings:**

Evidence highlights an increase in behavioral sleep intervention research targeting the sleep health of neurodivergent children. In addition to the traditional behavioral sleep intervention components, these interventions target neurodivergent-specific experiences that impact sleep difficulties like sensory processing differences, medication effects, and familial supports. Recent evidence affirms that behavioral interventions improve sleep health and positively impact daytime functioning for neurodivergent children.

**Summary:**

Behavioral interventions developed or adapted for neurodivergent children to target specific challenges they experience have emerging efficacy. Future research should aim to increase the generalizability of the preliminary findings with larger, more diverse samples and engagement with key community members to improve implementation success.

## Introduction

Sleep health is a critical component of growth and development in childhood. Sleep health includes the domains of sleep satisfaction, daytime alertness, sleep timing, sleep efficiency, and sleep duration [1]. Behaviors surrounding bedtime, such as parent-child interactions, common bedtime routines, and the stability of sleep patterns are also included in the pediatric sleep health definition [2]. Poor sleep health can have serious consequences for daytime functioning, physical and mental health, family functioning, and social-emotion development for children [3–6]. Specifically, poor sleep health can lead to decreased impulse control [7], increased reactivity [8], elevated depressive symptoms [9], and impaired executive functioning [6]- all characteristics related to several core symptoms of different neurodivergent diagnoses [10]. Poor sleep health has been reported for 60–95% of neurodivergent children [11–14]. Families of neurodivergent children report sleep health concerns like difficulty falling asleep, maintaining sleep, obstructive sleep apnea, and daytime fatigue. Therefore, researchers and clinicians are seeking to better understand and improve sleep health for neurodivergent children and their families.

*Neurodivergence* is a term that refers to processing styles that diverge from the majority *neurotypical* mind [15, 16]. Neurodivergence is often used to describe individuals who have diagnoses of attention deficit hyperactivity disorder (ADHD), autism, Down syndrome, learning disorders, depression, and anxiety, among others. The neurodiversity movement has shifted away from the medical model of disability, where person-first terminology (e.g., child with autism) is used to *identify-first* terminology (e.g., autistic person) for groups that prefer this approach [17]. As such, throughout this article, identify-first language will be used when describing research with autistic individuals and person first language will be used when describing research with other neurodiverse groups (e.g., children with ADHD).

Behavioral sleep interventions are commonly the first recommended line of defense when addressing poor sleep health. Pediatric behavioral sleep interventions often are rooted in cognitive behavioral therapy for insomnia (CBT-I) and are implemented within the family unit with engagement of the caregiver. CBT-I elements include caregiver and child education, cognitive strategies for relaxation, and behavioral strategies like stimulus control, sleep compression or restriction, bedtime routines, and caregiver-child interaction support [18]. Some interventions also include chronotherapy elements like bright light exposure and circadian rhythm education [19]. In a recent scoping review of 80 studies involving typically developing children, Dr. Meltzer and colleagues found that sleep hygiene, parent education, and relaxation techniques are common behavioral sleep intervention components for school-aged children [20].

In recent years, behavioral sleep interventions have been trialed in neurodiverse populations [21–24]. Some interventions require adaptations to target specific sleep difficulties experienced by neurodivergent children while others are implemented without adaptations. Research in this area is growing, however there is a gap in the advancement and implementation of behavioral sleep interventions that are tailored to neurodivergent children. The aim of this review is not to be exhaustive of all behavioral sleep interventions tested in neurodiverse populations, but to examine the most recent literature to identify new interventions developed and adapted for neurodiverse children and examine the targeted outcomes for school aged children. To achieve this, we reviewed behavioral sleep intervention research with neurodiverse children published in the past 5 years (2020–2025) using online databases (e.g., PubMED and Google Scholar). We also sought out related on-going NIH funded research using the NIH RePORTER.

## Unique Considerations for the Neurodiverse Populations

**Fig. 1:**
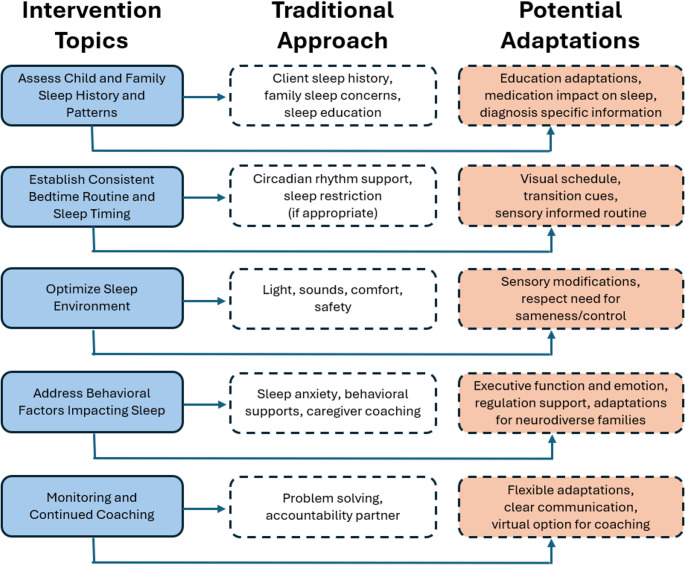
Intervention topics, traditional approaches, and potential adaptations for neurodiverse groups

Examining the unique aspects of sleep health for neurodivergent children is critical when adapting existing or developing new sleep interventions. While research in this area is on-going, recent studies have uncovered a number of critical considerations for intervention development and outcome measurements that will drive future research and medical care. In Fig. 1, we have highlighted specific adaptations that have been supported through research in neurodiverse populations.

### Disruptions in Circadian Rhythms

Circadian rhythms are biorhythms that have approximately 24-hour cycles that play an essential role in regulating various physiological processes in the body, namely sleep-wake and hormone cycles [25]. Some neurodivergent children who have sleep onset insomnia may have a circadian dysfunction where their dim light melatonin onset (DLMO) is significantly delayed and cortisol rhythms are blunted or delayed [13, 26–31]. For these individuals, chronotherapy interventions should be used to shift the circadian rhythm to better match the desired sleep timing. Melatonin supplementation (3–6 mg nightly) has been noted to support advancement of DLMO by 44 min across 4 weeks of intervention for medication-free children with ADHD and chronic sleep-onset insomnia compared with a placebo-control group who had a 13 min delay [32]. Research on the effects of bright light therapies to improve circadian rhythm dysfunction is limited in children, but has been found to be effective for adults with ADHD [31]. Interventions focused on promoting consistent sleep routines, exposure to natural light, and optimizing the sleep environment also address circadian rhythm disruptions and can improve sleep-onset insomnia [30].

### Medication Management and Effects

Neurodivergent children often take medication to manage daytime challenges and symptoms from co-morbid diagnoses, however sometimes these medications can impact sleep (e.g., stimulants or SSRIs to manage associated symptoms like irritability, hyperactivity, and anxiety). Medication use for daytime functioning was elevated for children with ADHD in two systematic reviews, with commonly reported use of stimulants (77% of participants across 10 studies) [21, 33]. Methylphenidate (MPH) in particular has been noted to be a first-line medication for ADHD, however long-term use is limited by low adherence and tolerability rates [34]. Similar elevated rates of medication were found for autistic children in a review of autistic youth who were enrolled in Medicaid between 2008–2016 [35]. This study examined rates of psychotropic prescriptions filled and found that 44–54% of autistic youth filled at least one psychotropic prescription across that time period, with common medications being antipsychotics (22%-36%), stimulants (20%-21%), antidepressants (19%-20%), and anticonvulsants (13%-17%).

Anecdotally, caregivers of children with ADHD within our studies recount through qualitative interviews long medication journeys that include balancing stimulants for daytime functioning and melatonin or α-Agonists for sleep onset (*unpublished*, Klingenstein Third Generation Foundation Fellowship grant 2024, PI Hartman). Future research is needed to confirm this observation and examine how best to support families through this process.

Often clinicians treating sleep concerns for neurodiverse children tend to rely on personal experience and expert opinion when choosing medication management for sleep disturbances [36]. However, emerging research has examined pharmacological treatments for sleep concerns in neurodivergent youth. A low dose [34] and immediate release [32, 37] melatonin have been found to significantly support increased sleep duration for children with ADHD. Melatonin + bright light therapy has been found to be an effective treatment for advancing DLMO for adults with ADHD [38]. Melatonin has also been found to increase total sleep time and decrease sleep onset latency as measured by actigraphy for autistic children [39]. Other medications like antihistamines and α-Agonists are also considered during treatment [36, 40]. Longer patch wear times of methylphenidate led to marginally significant improvements in sleep quality for a small sample of children with ADHD [41]. Importantly, two RCTs found that eszopiclone [42] and zolpidem [43] did not support improvements in latency to persistent sleep measured by polysomnography for children with ADHD.

Less has been published related to sleep medication efficacy in other neurodiverse populations. In a study of quality of life of caregivers of children with Down Syndrome and sleep problems, Fullwood and colleagues notes that sleep concerns are often related to sleep disordered breathing where treatments are adenotonsillectomy and continuous positive airway pressure- CPAP [24]. For non-respiratory sleep disorders, pharmacological treatments and behavioral interventions are under-researched for children with Down Syndrome.

### Sensory Processing Differences

Unique sensory processing can also influence sleep health for neurodivergent children. Sensory processing, or the way the brain recognizes, processes, and acts on sensory input from the environment and within the body, can be different for many neurodivergent children compared to peers [44]. Rates of sensory processing differences that have a significant impact on daily life range from approximately 50% of children with Down Syndrome, 50% of children with ADHD, and up to 90% of autistic children [45–47]. Sensory processing differences are highly heterogeneous and have been associated with sleep difficulties in multiple neurodiverse populations [48–50]. These difficulties include delayed sleep onset, sleep anxiety, bedtime resistance, night awakenings, and short sleep durations. Some hypothesize that sensory over-responsivity, also known as sensory sensitivities, specifically accounts for several sleep problems reported in neurodiverse populations [50]. Others report that tactile over/under responsivity or auditory over-responsivity are strongly associated with sleep difficulties in some neurodivergent groups [51, 52]. While research continues to test the specific aspects of sensory processing that impacts sleep, caregivers of children with sensory processing differences often describe general overstimulation and elevated arousal levels at bedtime as drivers of the poor child and family sleep health. Supports for sensory processing differences can include environmental adaptation to decrease sensory stimuli, incorporating sensory stimuli that is known to be calming within the bedtime routine, and involving an occupational therapist to support sensory processing and sleep needs. More discussion on sensory-specific interventions is highlighted within the *Active ingredients of intervention* section of this paper.

### Supports for Predictability and Transitions

In addition to sensory processing differences, neurodivergent children may find comfort in predictability and need support through transitions. Using tools like visual schedules and embedding behavioral sleep strategies within existing routines can be helpful for behavior change, as seen in Caldwell’s work on family meal behaviors with children with Down syndrome [53]. In this study, Caldwell and colleagues found that as parents integrated behavior strategies into their existing family meal routine, child behavior improved. Others have embedded sleep intervention tools (e.g., visual schedules) within existing routines that require minimal environmental changes and found this approach to be successful for neurodivergent families [54].

### Co-Occurring Diagnoses

Another consideration for neurodiverse groups is the common co-occurrence of diagnoses. For example, ADHD and autism have significant overlap in symptoms: 30–80% of autistic children also exhibit ADHD characteristics and 20–50% of children with ADHD have autism related characteristics [55, 56]. Therefore, considerations are needed that account for the complex interaction of behaviors associated with each specific diagnosis. For example, a child with ADHD and autistic characteristics may benefit from strategies designed to promote sleep health for autistic children, such as maintaining a predictable routine. Whereas a child with ADHD without autistic characteristics may be more flexible with their routines and potential disruption to those routines. Another example could be when considering treatment for a child with Down syndrome and autism, as opposed to a child with Down syndrome only, it is important for clinicians to know that a subset of autistic children may synthesize melatonin differently resulting in low melatonin levels [57]. A holistic view of each child and their specific diagnostic profile is needed to identify the most comprehensive intervention approach to promote sleep health. Flexible sleep interventions, like interventions that are modular, are supportive of this tailored approach.

### Family Neurodivergence

Given strong heritable components of neurodevelopmental conditions, it is important to acknowledge there is a high probability that there may be other members of the family who are neurodivergent [58, 59]. There is much to learn about how family neurodivergence interacts with the child’s sleep health and how behavioral interventions are impacted. Beyond the impact of family neurodivergence on child sleep, poor sleep health of the family members has its own health consequences. Parents with ADHD and their partners present with poorer sleep quality than those without ADHD and this poor sleep quality is associated with parenting stress [60]. Research in families raising autistic children documents biological parents with subclinical elements of autism have elevated rates of sleep problems that are distinct from their child’s sleep concerns [61, 62]. As a clinical example, a mother who participated in our on-going pilot study of a new intervention called *The Power Down Program* (NCT06406309, supported by the *Klingenstein Third Generation Foundation fellowship)* mentioned her own experience with ADHD makes it difficult for her to initiate bedtime at a consistent time each night in a semi-structured interview examining current bedtime challenges. She shared that often she notices that it is past the time she meant to start bedtime and then rushes the child through their bedtime routine which, admittedly, increases the child’s arousal level and results in a frustrating bedtime experience for all. Another parent who identifies as autistic shared in their semi-structured interview that they often find themselves “shutting down” around bedtime because it has been so stressful in the past. This impacts their ability to emotionally be present for their child during the transition of bedtime. Anecdotal evidence such as these stories imply that treatment modules focused on support for parents may be necessary for a successful behavioral sleep intervention among neurodivergent families, although further examination is needed to confirm these observations.

## Active Ingredients of Intervention 

Behavioral sleep interventions for neurodivergent children generally follow broad pediatric behavioral intervention themes: stimulus control, or associating the bed with sleep only; sleep restriction (adjusting bedtime to match more closely actual sleep onset time) or sleep compression (reducing time in bed prior to sleep onset); supporting a robust circadian rhythm and homeostatic sleep drive through regular routines, attention to light exposure timing, limiting caffeine and foods with simulant-like effects (e.g., chocolate); caregiver and child education on sleep hygiene, environmental adaptations to support sleep; behavior management to support caregiver-child interactions at bedtime, positive reinforcement; and cognitive strategies for sleep anxiety [20, 63]. In a recent review of 9 randomized control trials that included neurodiverse groups (autism, ADHD, and Down syndrome) from 1999 to 2022, they identified common intervention content to be behavior-based with parent coaching, cognitive-behavioral training, sleep hygiene education, and relaxation training [64]. Most interventions were manualized, delivered with caregiver involvement, and ranged from 1 to 12 sessions. There is also a recent exploration of possible inherent circadian disruption for some neurodiverse groups, which leads to circadian-based interventions (e.g., bright light therapy) [29].

### Caregiver Coaching and Family Education

Caregiver coaching and family education on sleep health are common components of behavioral sleep interventions. As such, caregiver coaching is increasingly included in interventions adapted for neurodiverse families [65]. Coaching sessions focus on diagnosis specific sleep information, the child’s sleep patterns, and ways to develop healthy sleep practices within their family unit, including behavior change approaches based on principles of reinforcement and extinction [66]. Some interventions tailor the order of information or topic of the coaching sessions to align with the caregiver’s most pressing concerns [67]. Topics can also address common sleep-related behavioral concerns for neurodiverse groups, like setting bedtime routines, limit setting for caregivers, cognitive strategies for relaxation, and how nutrition and the environment impact sleep [54, 68, 69]. Coaching sessions range in frequency and duration and can be conducted in a face-to-face setting or hybrid (telehealth and in person). As an example of a coaching-focused intervention, *Sleeping Sound in ASD*, paired caregiver coaching and child education with an individualized sleep management plan delivered through two 50-minute in-person sessions 2-weeks apart with a follow-up phone call. This intervention showed moderate to large effects of improvement in sleep related outcomes 3 months post randomization (per the Children’s Sleep Habits Questionnaire- caregiver report) [68]. In another study of children with ADHD, El-Monshed and colleagues combined sleep and ADHD-related education modules with child and caregiver training on sleep management approaches in their 10 session hybrid (5 in-person and 5 virtual sessions) intervention [54]. The intervention incorporated strategies that aligned with the family situation (e.g., addressed family valued goals) and considered the learning style of the child by leveraging visual strategies, social scripts, and timetables to strengthen the learning process. Improvement in caregiver-reported sleep (e.g., daytime sleepiness, bedtime resistance, sleep anxiety) and ADHD characteristics (e.g., hyperactivity, inattention) were noted (effect sizes of 1.9 and 1.8 respectively).

### Sensory-Based Interventions

Building on sensory processing differences in neurodiverse groups, interventions can employ sensory theories to support sleep health. As mentioned above, a new intervention called the *Power Down Program* uses caregiver-delivered moderate pressure massage and mindfulness techniques to help the child settle down at night. Preliminary findings document significant improvements in overall sleep (per caregiver reported Children’s Sleep Habits Questionnaires) after only two weeks of intervention for children with ADHD [70]. Ongoing studies are evaluating this intervention for autistic children and their families at a larger scale (Sleep Research Society Foundation Grant-NCT06386029, NIH K23HD119249-NCT07476937). Dr. Geela Spira has also trialed caregiver-delivered moderate massage to support sleep health for children with sensory processing differences in Isreal and noted significant improvements in multiple sleep health domains (CSHQ). Additionally, parents reported a significant improvement in time to fall asleep for children who received the massage intervention compared to those who did not (per sleep diary) [71]. Other research has piloted sensory-based tools to support sleep for neurodivergent children, like Touchpoints ™ [72] and weighted blankets [73]. In the small pilot study trialing Touchpoints™, a wearable device worn on the ankle that delivers gentle vibrations to decrease stress, children with sensory over-responsivity experienced significant improvements in sleep latency (actigraphy based) and parent reported improved sleep quality (Child Sleep Wake Scale [74]; going to bed, maintaining sleep, and overall score improved). Sleep duration, efficiency, or nighttime awakenings were not impacted. Weighted blankets have been studied as tools to improve sleep and anxiety across many populations [75]. In a cross-over RCT of 94 children with ADHD, Lönn and colleagues found moderate improvement in the actigraphy variables of total sleep time, wake after sleep onset, sleep efficiency, but not sleep onset latency during the use of the weighted blanket compared to a lighter “control” blanket [73].

### Physical Activity and Mindfulness

Physical activity and mindfulness interventions are also used to address sleep difficulties for neurodivergent children. These interventions include water-based aerobics [76–80], mind-body activities (e.g., yoga, karate, judo) [81–83], land-based aerobics (e.g., cycling, jogging, and basketball) [84–90]. Interestingly, in a systematic review and meta-analysis examining the relationship between physical activity interventions and sleep changes for children and adolescents with neurodevelopmental disorders, associations were only documented across studies using objective sleep measures (e.g., actigraphy) [91]. Distinct differences were found when comparing findings using subjective measures (caregiver reported questionnaires reported improvement in sleep disturbances and night wakings but not sleep duration or sleep resistance) and actigraphy measures (evident sleep efficiency and duration improvements but no significant changes in wakefulness after sleep onset). To note, the studies in this review were heterogeneous and had small sample sizes which may impact generalizability of these findings.

Interestingly, some studies examine pharmacological intervention paired with physical activity interventions to improve sleep for neurodiverse children. One such study compares a cycling intervention to melatonin supplementation for autistic children in a randomized clinical trial. Similar improvements across groups in sleep parameters (efficiency, onset latency, and duration) for participants in the cycling, melatonin, and combined groups compared to a placebo control group were found [92].

## Outcomes of Interest

Outcomes in sleep intervention research broadly focus on improving sleep health domains: sleep duration, efficiency, satisfaction, behaviors, daytime functioning, and nighttime awakenings. Intervention studies in neurodiverse populations often rely on a combination of subjective (caregiver-reported questionnaires and sleep diaries) and movement-based (actigraphy) outcome measures to assess change in sleep health. Broadly, subjective measures tend to be used more frequently. As an example, in a recent systematic review of 15 unique sleep interventions for children with neurodevelopmental and medical conditions, 6 studies used both questionnaires and actigraphy, 6 more used questionnaires alone, 2 used actigraphy alone, and one used sleep diaries alone [64]. Questionnaires used included the Children’s Sleep Habits Questionnaire [93], the Sleep Disturbances Scale for Children [94], and PROMIS proxy sleep measures [95]. Common actigraphy outcomes include sleep efficiency, sleep onset latency, total sleep time, and wake after sleep onset [64, 96]. Interestingly, decreasing sleep onset latency tends to be the target of behavioral sleep interventions, however evidence suggests that beyond the traditional sleep onset latency variable there are novel characteristics of the settling down time period that should be considered [97]. In a secondary analysis of actigraphy data, Kocanaogullari and colleagues applied machine learning to examine characteristics of the settling down period, or the time during which a child is trying to fall asleep prior to first sleep onset, that were unique for children with sensory over-responsivity [98]. They found that activity characteristics, namely maximum magnitude of activity, during the settling down period was predictive of the group, meaning children with sensory over-responsivity tended to be more active during the settling down period, perhaps impacting traditional sleep onset latency measures. Future research is needed to validate actigraphy variables for neurodiverse children who may settle to sleep or self-soothe (e.g., rocking) in a way that is not characterized traditionally in actigraphy.

In addition to traditional sleep health outcomes of interest, some behavioral sleep interventions assess change in symptomology or characteristics that impact daily life. For example, in a review of sleep interventions for children with ADHD, some studies included measures of health-related quality of life, school attendance, working memory tests, strengths and difficulties questionnaire [99], and ADHD symptoms as outcomes of interest [21, 33]. In a systematic review examining effects of behavioral sleep interventions with autistic children, improvements on daytime functioning were found including reduction in stereotypic behaviors, improvement in emotion regulation, and improvement in quality of life [100]. As research continues to explore the effects of sleep interventions for neurodiverse groups, outcome measures of emotion regulation, educational attainment, family functioning, and daytime functioning should continue to be examined.

## Delivery Methods and Settings for Behavioral Sleep Intervention

The delivery method and setting of behavioral sleep interventions can provide key adaptations for neurodivergent children and their families. Researchers highlight adaptations to employ sleep interventions virtually can assist with reaching those with limited access to care [101]. These adaptations can be especially supportive for families of neurodivergent children who may have additional barriers like various appointments, difficulties with transitions and change, and overall complex lives [102].

While some interventions use the traditional method of individual, in-person sessions, new interventions employ a hybrid, group, or virtual/telehealth delivery. Hybrid interventions use the in-person sessions to set goals and create a tailored sleep management plan and virtual sessions to check in with the family, problem solve, and answer questions that come up as the family implements the changes. While hybrid interventions are less common, emerging evidence suggests that this modality can be effective for neurodivergent families, with improvement in sleep and quality of life noted after pilot trials [54].

Telehealth or eHealth interventions are also being adapted to meet the needs of neurodivergent families. Corkum and colleagues adapted the eHealth intervention called *Better Nights*,* Better Days* for neurodivergent children (BNBD-ND), specifically children with ADHD, autism, cerebral palsy, and fetal alcohol spectrum disorder. The original BNBD program was delivered through 5, self-guided, online sessions. Through an iterative process, the BNBD-ND intervention was adapted to addresses comorbidities often experienced by neurodiverse children and allows for tailoring of the program for each child and family, while keeping the eHealth model [103]. Continued work is being done to refine this intervention and address identified barriers to engagement [104].

In-person intervention sessions in an outpatient setting are common for behavioral sleep interventions. The *Transdiagnostic Sleep and Circadian Intervention* (TSC) is an example of one such program that is modular and is delivered using 4 cross cutting “core” modules and 4 option modules in sessions lasting 50–60 min [105, 106]. The TSC was developed to be used with at-risk adolescents or adults with severe mental illness and sleep problems. Recently, the TSC has been trialed without adaptations with adolescents with ADHD and was found to be feasible, acceptable, and effective in improving overall sleep and daytime sleepiness (both adolescent and parent reported) [107]. In a Danish randomized controlled trial, the TSC was trialed with young adults with ADHD, bipolar, or depression in an outpatient setting with a multidisciplinary team including a nurse, physiotherapist, occupational therapist, mental health educator, and psychologist [108]. This trial found that the TSC resulted in improved sleep quality, reduced insomnia severity, and increased well-being and health (measured through questionnaires, sleep diaries, and actigraphy) when compared to a single session of sleep hygiene education. Adaptations of the TSC are underway to address sleep and circadian disruption in autistic adults. Autistic community partners, care partners, and clinicians identified adjustments like (1) adapting content to simplify components and increase memory supports and (2) enhancing delivery (e.g., offer in-person and telehealth options, increase check-ins between sessions, increase number of sessions) to best support autistic individuals [109].

There are many documented provider-, patient-, and system-level barriers to care for individuals with insomnia or insufficient sleep health [110]. One significant barrier is access to trained healthcare professionals who can deliver sleep interventions. Often, behavioral sleep interventions are delivered by psychologists and mental health professionals. For neurodivergent children, this means an additional therapy appointment in addition to other services they might receive like occupational, physical, and speech therapy. There is a push for more occupational therapists to address sleep health concerns within the many settings they serve [111]. Occupational therapists are embedded in outpatient, inpatient, school, and home settings and often support their clients with weekly therapy sessions. Their expertise in sensory processing, routine establishment, behavioral interventions, parent coaching, habit training, environmental adaptations, and family system dynamics makes occupational therapists well-suited to be sleep champions for neurodivergent children.

## Future Research Opportunities and Upcoming Results

Behavioral sleep intervention research has had promising advances in neurodiverse populations over the past 10 years. While recent publications, in large part, focus on small sample sizes piloting new interventions, there is a consensus that targeting sleep in neurodiverse youth improves sleep and daily functioning. In general, child-focused neurodiversity research has included majority male participants and infrequently reported ethnicity and race of the sample [112]. As research in this focus area continues to grow, we encourage researchers to increase female and gender-diverse representation and expand the racial, ethnic, and socioeconomic diversity in larger samples to evaluate the acceptability and generalizability prior to larger implementation. We also see opportunities to explore different settings for sleep intervention implementation, such as within primary care clinics, outpatient occupational therapy sessions, or schools. Additionally, more work is needed to understand the unique sleep health targets for neurodivergent youth and potential benefits of behavioral sleep interventions on outcomes such as cognition, academic skills, emotion dysregulation, sensory processing skills, parent sleep, and family sleep health.

We are currently in a time of growth for this important area of research, with many interventions in the development and refinement phases. In the near future, expect to see results from Malkani and colleagues’ study comparing in-person versus online delivery of the *Sleeping Sound* behavioral sleep intervention for children with ADHD [113]. The *Better Nights*,* Better Days* for neurodivergent children intervention is also being tested for large scale implementation using their self-guided eHealth platform [104]. A new preschool-aged sleep intervention protocol, called the *Preschool Attention and Sleep Support* (PASS), was recently published prior to a feasibility randomized clinical trial comparing the PASS to behavioral parent training [114]. Also in preschool-aged children, Joseph and colleagues are testing a sleep-focused parent behavioral intervention for preschool children with elevated ADHD symptoms and behavioral sleep symptoms within the primary care setting [115]. Additionally, final analyses are being conducted from the *Power Down Program* pilot study for children with ADHD and on-going studies are piloting the program with autistic children and their families. A new protocol was also published outlining a randomized controlled trial testing the efficacy of the *Sleep IntervEntion as Symptom Treatment for ADHD (SIESTA)* to improve sleep, ADHD symptoms, and related problems for adolescents with ADHD [116].

On-going studies related to understanding unique contributors to poor sleep health for neurodiverse groups will narrow the gap of knowledge and inform future intervention development and adaptation. Some on-going NIH-supported behavioral intervention trials include Hartman and colleagues’ study probing the role of sensory regulation in sleep health and emotion dysregulation for autistic youth (Project number K23HD119249), Carskadon and colleagues’ studies from their COBRE center for sleep and circadian rhythms in child and adolescent mental health (P20GM139743-03), and Acosta and colleagues’ examination of parenting practices and their effect on adolescent sleep health (F31HD101257). Additionally, Johnson and colleagues are building on their *Sleep Parent Training* pilot study through an on-going multi-site randomized controlled trial of their parent-mediated telehealth intervention with autistic children and their caregivers (R01HD114631).

## Conclusion

In this review, we summarized reports published in the past 5 years focused on new behavioral sleep interventions developed or adapted for neurodivergent children. Many studies present sleep interventions rooted in gold-standard sleep intervention theories that have been adapted to address unique concerns that neurodivergent children and their families experience related to sleep health. Findings from on-going studies are promising and demonstrate significant and clinically meaningful improvements in sleep health and daytime outcomes. As sleep research continues to expand in neurodiverse populations and our understanding of the unique contributors to elevated poor sleep improves, clinicians will have more tailored sleep interventions to use in the clinic.

## Key References

- Phillips NL, Moore T, Teng A, Brookes N, Palermo TM, Lah S. Behavioral interventions for sleep disturbances in children with neurological and neurodevelopmental disorders: a systematic review and meta-analysis of randomized controlled trials. Sleep. 2020 Sep 14;43(9):zsaa040. doi: 10.1093/sleep/zsaa040. PMID: 32163581.
  - ○ This article reviews the more recent RCTs of behavioral sleep interventions with neurodivergent children.
- Santos RA, Costa LH, Linhares RC, Pradella-Hallinan M, Coelho FMS, Oliveira GdP. Sleep disorders in Down syndrome: a systematic review. Arq Neuropsiquiatr. 2022;80(04):424 − 43. doi: 10.1590/0004-282X-ANP-2021-0242.
  - ○ This article provides a review of research in sleep disorder for people with Down syndrome.
- Larsson I, Aili K, Lönn M, Svedberg P, Nygren JM, Ivarsson A, et al. Sleep interventions for children with attention deficit hyperactivity disorder (ADHD): A systematic literature review. Sleep Med. 2023;102:64–75. doi: 10.1016/j.sleep.2022.12.021.
  - ○ This article is a review of sleep interventions for children with ADHD.
- Kamara D, Bernard A, Clark EL, Duraccio KM, Ingram DG, Li T, et al. Systematic review and meta-analysis of behavioral interventions for sleep disruption in pediatric neurodevelopmental and medical conditions. Journal of pediatric psychology. 2025:jsae096.
  - ○ This article reviews behavioral interventions across multiple diagnostic groups, including ADHD, autism, and Down syndrome*.*

## Data Availability

No datasets were generated or analysed during the current study.

## References

[1] Buysse DJ. Sleep health: can we define it? Does it matter? Sleep. 2014;37(1):9–17. 10.5665/sleep.3298. 24470692 10.5665/sleep.3298 PMC3902880

[2] Meltzer, Williamson. Mindell. Pediatric sleep health: It matters, and so does how we define it. Sleep Med Rev. 2021;57:101425. 10.1016/j.smrv.2021.101425. 33601324 10.1016/j.smrv.2021.101425 PMC9067252

[3] Matricciani L, Paquet C, Galland B, Short M, Olds T. Children’s sleep and health: A meta-review. Sleep Med Rev. 2019;46:136–50. 10.1016/j.smrv.2019.04.011. 31121414 10.1016/j.smrv.2019.04.011

[4] Schlieber M, Han J. The Role of Sleep in Young Children’s Development: A Review. J Genet Psychol. 2021;182(4):205–17. 10.1080/00221325.2021.1908218. 33825621 10.1080/00221325.2021.1908218

[5] Lollies F, Schnatschmidt M, Schlarb AA, Genuneit J. Child Sleep Problems Affect Mothers and Fathers Differently: How Infant and Young Child Sleep Affects Paternal and Maternal Sleep Quality, Emotion Regulation, and Sleep-Related Cognitions. Nat Sci Sleep. 2022;14(null):137–52. 10.2147/NSS.S329503 10.2147/NSS.S329503 PMC8801371 35115855

[6] Liu J, Ji X, Pitt S, Wang G, Rovit E, Lipman T, et al. Childhood sleep: physical, cognitive, and behavioral consequences and implications. World J Pediatr. 2024;20(2):122–32. 10.1007/s12519-022-00647-w. 36418660 10.1007/s12519-022-00647-w PMC9685105

[7] Guarana CL, Ryu JW, O’Boyle EH, Lee J, Barnes CM. Sleep and self-control: A systematic review and meta-analysis. Sleep Med Rev. 2021;59:101514. 10.1016/j.smrv.2021.101514. 34157493 10.1016/j.smrv.2021.101514

[8] Sesso G, Guccione F, Pisano S, Valente E, Narzisi A, Berloffa S, et al. Emotional dysregulation and sleep problems: a transdiagnostic approach in youth. Clin Pract. 2024;14(3):934–45. 38804406 10.3390/clinpract14030074 PMC11130951

[9] Becker SP, Tamm L, Epstein JN, Beebe DW. Impact of sleep restriction on affective functioning in adolescents with attention-deficit/hyperactivity disorder. J Child Psychol Psychiatry. 2020;61(10):1160–8. 10.1111/jcpp.13235. 32157691 10.1111/jcpp.13235 PMC7483709

[10] Association AP. Diagnostic and statistical manual of mental disorders (5th ed.). Am Psychiatric Assoc. 2013.

[11] Santos RA, Costa LH, Linhares RC, Pradella-Hallinan M, Coelho FMS, Oliveira GP. Sleep disorders in Down syndrome: a systematic review. Arq Neuropsiquiatr. 2022;80(04):424–43. 10.1590/0004-282X-ANP-2021-0242. 35293557 10.1590/0004-282X-ANP-2021-0242 PMC9173224

[12] Chawla JK, Burgess S, Heussler H. The impact of sleep problems on functional and cognitive outcomes in children with Down syndrome: a review of the literature. J Clin Sleep Med. 2020;16(10):1785–95. 32536364 10.5664/jcsm.8630 PMC7954012

[13] van der Heijden KB, Stoffelsen RJ, Popma A, Swaab H. Sleep, chronotype, and sleep hygiene in children with attention-deficit/hyperactivity disorder, autism spectrum disorder, and controls. Eur Child Adolesc Psychiatry. 2018;27(1):99–111. 10.1007/s00787-017-1025-8. 28689312 10.1007/s00787-017-1025-8 PMC5799342

[14] Malow BA, Katz T, Reynolds AM, Shui A, Carno M, Connolly HV, et al. Sleep Difficulties and Medications in Children With Autism Spectrum Disorders: A Registry Study. Pediatrics. 2016;137(Supplement2):S98–104. 10.1542/peds.2015-2851H. 26908483 10.1542/peds.2015-2851H

[15] McGee M. Neurodiversity Contexts. 2012;11(3):12–3.

[16] Armstrong T, Neurodiversity. A concept whose time has come. American Institute for Learning and Development. https://www.institute4learningcom/resources/articles/neurodiversity. 2010.

[17] Bury SM, Jellett R, Spoor JR, Hedley D. It Defines Who I Am or It’s Something I Have: What Language Do [Autistic] Australian Adults [on the Autism Spectrum] Prefer? J Autism Dev Disord. 2020. 10.1007/s10803-020-04425-3. 10.1007/s10803-020-04425-3 32112234

[18] Walker J, Muench A, Perlis ML, Vargas I. Cognitive Behavioral Therapy for Insomnia (CBT-I): A Primer. Klin Spec Psihol. 2022;11(2):123–37. 10.17759/cpse.2022110208. 36908717 10.17759/cpse.2022110208 PMC10002474

[19] van Andel E, Bijlenga D, Vogel SW, Beekman AT, Kooij JS. Attention-deficit/hyperactivity disorder and delayed sleep phase syndrome in adults: A randomized clinical trial on the effects of chronotherapy on sleep. J Biol Rhythm. 2022;37(6):673–89. 10.1177/07487304221124659 36181304

[20] Meltzer LJ, Wainer A, Engstrom E, Pepa L, Mindell JA. Seeing the whole elephant: a scoping review of behavioral treatments for pediatric insomnia. Sleep Med Rev. 2021;56:101410. 33387973 10.1016/j.smrv.2020.101410

[21] Malkani MK, Pestell CF, Sheridan AMC, Crichton AJ, Horsburgh GC, Bucks RS. Behavioral Sleep Interventions for Children With ADHD: A Systematic Review and Meta-Analysis. J Atten Disord. 2022;26(14):1805–21. 10.1177/10870547221106239. 35758199 10.1177/10870547221106239

[22] Hornsey SJ, Gosling CJ, Jurek L, Nourredine M, Telesia L, Solmi M, et al. Umbrella Review and Meta-Analysis: The Efficacy of Nonpharmacological Interventions for Sleep Disturbances in Children and Adolescents. J Am Acad Child Adolesc Psychiatry. 2024. 10.1016/j.jaac.2024.10.015 39608635

[23] Esbensen AJ, Hoffman EK, Beebe DW, Byars K, Carle AC, Epstein JN, et al. Randomized Behavioral Sleep Clinical Trial to Improve Outcomes in Children With Down Syndrome. Am J Intellect Dev Disabil. 2022;127(2):149–64. 10.1352/1944-7558-127.2.149. 35180779 10.1352/1944-7558-127.2.149 PMC8867746

[24] Fullwood K, Thorpe K, Coles L, Vandeleur M, Waters K, Chawla J. Behavioural sleep interventions for the management of non-respiratory sleep disorders in children with neurodisability. Sleep Med Rev. 2025;102185. 10.1016/j.smrv.2025.102185 41202522

[25] Biological, Rhythms. Neuroscience.

[26] Van Veen MM, Kooij JJS, Boonstra AM, Gordijn MCM, Van Someren EJW. Delayed Circadian Rhythm in Adults with Attention-Deficit/Hyperactivity Disorder and Chronic Sleep-Onset Insomnia. Biol Psychiatry. 2010;67(11):1091–6. 10.1016/j.biopsych.2009.12.032. 20163790 10.1016/j.biopsych.2009.12.032

[27] Van der Heijden KB, Smits MG, Someren EJV, Boudewijn Gunning W. Idiopathic chronic sleep onset insomnia in attention-deficit/hyperactivity disorder: A circadian rhythm sleep disorder. Chronobiol Int. 2005;22(3):559–70. 16076654 10.1081/CBI-200062410

[28] Martinez-Cayuelas E, Gavela-Pérez T, Rodrigo-Moreno M, Losada-Del Pozo R, Moreno-Vinues B, Garces C, et al. Sleep problems, circadian rhythms, and their relation to behavioral difficulties in children and adolescents with autism spectrum disorder. J Autism Dev Disord. 2024;54(5):1712–26. 36869970 10.1007/s10803-023-05934-7 PMC9984759

[29] Bouteldja AA, Penichet D, Srivastava LK, Cermakian N. The circadian system: A neglected player in neurodevelopmental disorders. Eur J Neurosci. 2024;60(2):3858–90. 10.1111/ejn.16423. 38816965 10.1111/ejn.16423

[30] Giannotta G, Ruggiero M, Trabacca A. Chronobiology in paediatric neurological and neuropsychiatric disorders: harmonizing care with biological clocks. J Clin Med. 2024;13(24):7737. 39768659 10.3390/jcm13247737 PMC11678831

[31] Luu B, Fabiano N. ADHD as a circadian rhythm disorder: evidence and implications for chronotherapy. Front Psychiatry. 2025;16:1697900. 41450833 10.3389/fpsyt.2025.1697900 PMC12728042

[32] Van der Heijden KB, Smits MG, Van Someren EJW, Ridderinkhof KR, Gunning WB. Effect of Melatonin on Sleep, Behavior, and Cognition in ADHD and Chronic Sleep-Onset Insomnia. J Am Acad Child Adolesc Psychiatry. 2007;46(2):233–41. 10.1097/01.chi.0000246055.76167.0d. 17242627 10.1097/01.chi.0000246055.76167.0d

[33] Larsson I, Aili K, Lönn M, Svedberg P, Nygren JM, Ivarsson A, et al. Sleep interventions for children with attention deficit hyperactivity disorder (ADHD): A systematic literature review. Sleep Med. 2023;102:64–75. 10.1016/j.sleep.2022.12.021. 36603513 10.1016/j.sleep.2022.12.021

[34] Checa-Ros A, Muñoz-Hoyos A, Molina-Carballo A, Viejo-Boyano I, Chacín M, Bermúdez V, et al. Low Doses of Melatonin to Improve Sleep in Children with ADHD: An Open-Label Trial. Children. 2023;10(7):1121. 37508618 10.3390/children10071121 PMC10378280

[35] Rast JE, Tao S, Schott W, Shea LL, Brodkin ES, Kerns CM, et al. Psychotropic Medication Use in Children and Youth with Autism Enrolled in Medicaid. J Autism Dev Disord. 2025;55(1):258–66. 10.1007/s10803-023-06182-5. 38113012 10.1007/s10803-023-06182-5 PMC11228548

[36] Mammarella V, Orecchio S, Cameli N, Occhipinti S, Marcucci L, De Meo G, et al. Using pharmacotherapy to address sleep disturbances in autism spectrum disorders. Expert Rev Neurother. 2023;23(12):1261–76. 10.1080/14737175.2023.2267761. 37811652 10.1080/14737175.2023.2267761

[37] Weiss MD, Wasdell MB, Bomben MM, Rea KJ, Freeman RD. Sleep Hygiene and Melatonin Treatment for Children and Adolescents With ADHD and Initial Insomnia. J Am Acad Child Adolesc Psychiatry. 2006;45(5):512–9. 10.1097/01chi.0000205706.78818.ef. 16670647

[38] van Andel E, Bijlenga D, Vogel SWN, Beekman ATF, Kooij JJS. Attention-Deficit/Hyperactivity Disorder and Delayed Sleep Phase Syndrome in Adults: A Randomized Clinical Trial on the Effects of Chronotherapy on Sleep. J Biol Rhythm. 2022;37(6):673–89. 10.1177/07487304221124659. 10.1177/07487304221124659 36181304

[39] Nogueira HA, de Castro CT, da Silva DCG, Pereira M. Melatonin for sleep disorders in people with autism: Systematic review and meta-analysis. Prog Neuropsychopharmacol Biol Psychiatry. 2023;123:110695. 36584862 10.1016/j.pnpbp.2022.110695

[40] Cortese S, Fusetto Veronesi G, Gabellone A, Margari A, Marzulli L, Matera E, et al. The management of sleep disturbances in children with attention-deficit/hyperactivity disorder (ADHD): an update of the literature. Expert Rev Neurother. 2024;24(6):585–96. 38738544 10.1080/14737175.2024.2353692

[41] Ashkenasi A. Effect of Transdermal Methylphenidate Wear Times on Sleep in Children With Attention Deficit Hyperactivity Disorder. Pediatr Neurol. 2011;45(6):381–6. 10.1016/j.pediatrneurol.2011.09.003. 22115000 10.1016/j.pediatrneurol.2011.09.003

[42] Sangal RB, Blumer JL, Lankford DA, Grinnell TA, Huang H. Eszopiclone for Insomnia Associated With Attention-Deficit/Hyperactivity Disorder. Pediatrics. 2014;134(4):e1095–103. 10.1542/peds.2013-4221. 25266438 10.1542/peds.2013-4221

[43] Blumer JL, Findling RL, Shih WJ, Soubrane C, Reed MD. Controlled Clinical Trial of Zolpidem for the Treatment of Insomnia Associated With Attention-Deficit/ Hyperactivity Disorder in Children 6 to 17 Years of Age. Pediatrics. 2009;123(5):e770–6. 10.1542/peds.2008-2945. 19403468 10.1542/peds.2008-2945

[44] Salah A, Amr M, El-Sayed M, ElWasify M, Eltoukhy K, Salama S, et al. Sensory processing patterns among children with autism spectrum disorder (ASD) and attention deficit hyperactivity disorder (ADHD) using short sensory profile and evoked potentials: a case–control study. Middle East Curr Psychiatry. 2024;31(1):52. 10.1186/s43045-024-00441-6.

[45] McCormick C, Hepburn S, Young GS, Rogers SJ. Sensory symptoms in children with autism spectrum disorder, other developmental disorders and typical development: A longitudinal study. Autism. 2016;20(5):572–9. 10.1177/1362361315599755. 26395236 10.1177/1362361315599755 PMC4918912

[46] Panagiotidi M, Overton PG, Stafford T. The relationship between ADHD traits and sensory sensitivity in the general population. Compr Psychiatry. 2018;80:179–85. 10.1016/j.comppsych.2017.10.008. 29121555 10.1016/j.comppsych.2017.10.008

[47] Bruni M, Cameron D, Dua S, Noy S. Reported sensory processing of children with Down syndrome. Phys Occup Ther Pediatr. 2010;30(4):280–93. 10.3109/01942638.2010.486962. 20735195 10.3109/01942638.2010.486962

[48] Lane SJ, Leão MA, Spielmann V, Sleep. Sensory Integration/Processing, and Autism: A Scoping Review. Front Psychol. 2022;13. 10.3389/fpsyg.2022.877527. 10.3389/fpsyg.2022.877527 PMC9152214 35656493

[49] Hartman A, McKendry S, Soehner A, Bodison S, Akcakaya M, DeAlmeida D, et al. Characterizing sleep differences in children with and without sensory sensitivities. Front Psychol. 2022. 10.3389/fpsyg.2022.875766. 35814144 10.3389/fpsyg.2022.875766 PMC9257069

[50] Mimouni-Bloch A, Offek H, Engel-Yeger B, Rosenblum S, Posener E, Silman Z, et al. Association between sensory modulation and sleep difficulties in children with Attention Deficit Hyperactivity Disorder (ADHD). Sleep Med. 2021;84:107–13. 34144449 10.1016/j.sleep.2021.05.027

[51] Jamiol-Milc D, Bloch M, Liput M, Stachowska L, Skonieczna-Żydecka K. Tactile Processing and Quality of Sleep in Autism Spectrum Disorders. Brain Sci. 2021;11(3):362. 33808992 10.3390/brainsci11030362 PMC8001965

[52] Mazurek MO, Petroski GF. Sleep problems in children with autism spectrum disorder: examining the contributions of sensory over-responsivity and anxiety. Sleep Med. 2015;16(2):270–9. 10.1016/j.sleep.2014.11.006. 25600781 10.1016/j.sleep.2014.11.006

[53] Caldwell AR, Skidmore ER, Bendixen RM, Terhorst L. Examining child mealtime behavior as parents are coached to implement the Mealtime PREP intervention in the home: Findings from a pilot study. Br J Occup Therapy. 2020;83(10):631–7. 10.1177/0308022620920086. 10.1177/0308022620920086 PMC10254738 37304357

[54] El-Monshed AH, Loutfy A, El-Boraie H, El-Gilany AH, Fayed SM, Elzeiny A, et al. The efficacy of behavioral sleep intervention on sleep problems among children with attention-deficit hyperactivity disorder: A randomized controlled trial. J Nurs Scholarsh. 2025;57(3):380–93. 10.1111/jnu.13037. 39587035 10.1111/jnu.13037

[55] Murray MJ. Attention-deficit/hyperactivity disorder in the context of autism spectrum disorders. Curr psychiatry Rep. 2010;12(5):382–8. 20694583 10.1007/s11920-010-0145-3

[56] Van Der Meer JM, Oerlemans AM, Van Steijn DJ, Lappenschaar MG, De Sonneville LM, Buitelaar JK, et al. Are autism spectrum disorder and attention-deficit/hyperactivity disorder different manifestations of one overarching disorder? Cognitive and symptom evidence from a clinical and population-based sample. J Am Acad Child Adolesc Psychiatry. 2012;51(11):1160–72. e3. 23101742 10.1016/j.jaac.2012.08.024

[57] Melke J, Goubran Botros H, Chaste P, Betancur C, Nygren G, Anckarsäter H, et al. Abnormal melatonin synthesis in autism spectrum disorders. Mol Psychiatry. 2008;13(1):90–8. 17505466 10.1038/sj.mp.4002016 PMC2199264

[58] Uchida M, DiSalvo M, Walsh D, Biederman J. The heritability of ADHD in children of ADHD parents: a post-hoc analysis of longitudinal data. J Atten Disord. 2023;27(3):250–7. 36384349 10.1177/10870547221136251 PMC9969349

[59] Tick B, Bolton P, Happé F, Rutter M, Rijsdijk F. Heritability of autism spectrum disorders: a meta-analysis of twin studies. J Child Psychol Psychiatry. 2016;57(5):585–95. 26709141 10.1111/jcpp.12499 PMC4996332

[60] Joseph HM, Khetarpal SK, Wilson MA, Molina BSG. Parent ADHD Is Associated With Greater Parenting Distress in the First Year Postpartum. J Atten Disord. 2022;26(9):1257–68. 10.1177/10870547211066488. 34937412 10.1177/10870547211066488 PMC9098664

[61] El-Bouhali‐Abdellaoui F, Voltas N, Morales‐Hidalgo P, Canals J. Examining the Relationship Between Parental Broader Autism Phenotype Traits, Offspring Autism, and Parental Mental Health. Autism Res. 2025;18(2):387–401. 39713974 10.1002/aur.3295 PMC11826035

[62] Mannion A, Leader G. Relationship between child sleep problems in autism spectrum disorder and parent mental health and well-being. Sleep Med. 2023;109:4–10. 10.1016/j.sleep.2023.05.009. 37379630 10.1016/j.sleep.2023.05.009

[63] Pattison E, Papadopoulos N, Marks D, McGillivray J, Rinehart N. Behavioural treatments for sleep problems in children with autism spectrum disorder: A review of the recent literature. Curr psychiatry Rep. 2020;22(9):46. 32661719 10.1007/s11920-020-01172-1

[64] Kamara D, Bernard A, Clark EL, Duraccio KM, Ingram DG, Li T et al. Systematic review and meta-analysis of behavioral interventions for sleep disruption in pediatric neurodevelopmental and medical conditions. J Pediatr Psychol. 2025;jsae096. 10.1093/jpepsy/jsae096 39932204

[65] Kirkpatrick B, Louw JS, Leader G. Efficacy of parent training incorporated in behavioral sleep interventions for children with autism spectrum disorder and/or intellectual disabilities: A systematic review. Sleep Med. 2019;53:141–52. 30529483 10.1016/j.sleep.2018.08.034

[66] Ip BYT, Lee S-L, Li SX. Telehealth-delivered parent-based sleep-focused intervention for insomnia in preschool children with autism spectrum disorder: A randomized controlled study. Autism. 2024;28(11):2881–96. 10.1177/13623613241246502. 38725311 10.1177/13623613241246502

[67] McCrae CS, Chan WS, Curtis AF, Deroche CB, Munoz M, Takamatsu S, et al. Cognitive behavioral treatment of insomnia in school-aged children with autism spectrum disorder: A pilot feasibility study. Autism Res. 2020;13(1):167–76. 31566918 10.1002/aur.2204

[68] Papadopoulos N, Sciberras E, Hiscock H, Williams K, McGillivray J, Mihalopoulos C, et al. Sleeping Sound Autism Spectrum Disorder (ASD): A randomised controlled trial of a brief behavioural sleep intervention in primary school-aged autistic children. J Child Psychol Psychiatry. 2022;63(11):1423–33. 35285017 10.1111/jcpp.13590 PMC9790415

[69] Johnson CR, Barto L, Worley S, Rothstein R, Alder ML. Telehealth parent training for sleep disturbances in young children with autism spectrum disorder: A randomized controlled trial. Sleep Med. 2023;111:208–19. 10.1016/j.sleep.2023.08.033. 37806263 10.1016/j.sleep.2023.08.033

[70] Hartman A, Joseph H, Northrup J, Murray T, Brovender I, Wong V, et al. 1016 The Power Down: A New Sensory-based Bedtime Intervention for Children with ADHD. Sleep. 2025;48(Supplement1):A440–A. 10.1093/sleep/zsaf090.1016.

[71] Spira G. A sensory intervention to improve sleep behaviors and sensory processing behaviors of children with sensory processing disorders. Ir J Occup Therapy. 2021;49(1):11–20. 10.1108/IJOT-09-2020-0014.

[72] McGhee K, Kidney E, Pou K, Pruyn Bouley H, Reynolds S. The Effectiveness of Bilateral Alternating Tactile Stimulation for Improving Sleep in Children with Sensory over-Responsivity. Occup Therapy Health Care. 2021;35(4):424–41. 10.1080/07380577.2021.1946734. 10.1080/07380577.2021.1946734 34278921

[73] Lönn M, Svedberg P, Nygren J, Jarbin H, Aili K, Larsson I. The efficacy of weighted blankets for sleep in children with attention-deficit/hyperactivity disorder—A randomized controlled crossover trial. J Sleep Res. 2023;33(2):e13990. 10.1111/jsr.13990. 37452697 10.1111/jsr.13990

[74] LeBourgeois MK, Harsh JR. Development and psychometric evaluation of the Children’s Sleep-Wake Scale. Sleep Health. 2016;2(3):198–204. 10.1016/j.sleh.2016.04.001. 28066802 10.1016/j.sleh.2016.04.001 PMC5215091

[75] Bolic Baric V, Skuthälla S, Pettersson M, Gustafsson PA, Kjellberg A. The effectiveness of weighted blankets on sleep and everyday activities – A retrospective follow-up study of children and adults with attention deficit hyperactivity disorder and/or autism spectrum disorder. Scand J Occup Ther. 2023;30(8):1357–67. 10.1080/11038128.2021.1939414. 34184958 10.1080/11038128.2021.1939414

[76] Ansari S, AdibSaber F, Elmieh A, Gholamrezaei S. The effect of water-based intervention on sleep habits and two sleep-related cytokines in children with autism. Sleep Med. 2021;82:78–83. 33906043 10.1016/j.sleep.2021.03.045

[77] Lawson LM, Little L. Feasibility of a swimming intervention to improve sleep behaviors of children with autism spectrum disorder. Therapeutic Recreation J. 2017;51(2).

[78] Lawson LM, Kivlin N. Exploring the Effects of Swimming on Sleep Behaviors of Children with Autism Spectrum Disorder Using Single-Subject Design. Therapeutic Recreation J. 2022;56(4).

[79] Kanupka JW, Oriel KN, George CL, Crist L, Deardorff K, Douglass D, et al. The impact of aquatic exercise on sleep behaviors in children with Autism Spectrum Disorder. J Intellect Disability-Diagnosis Treat. 2018;6(1):1–7.

[80] Oriel KN, Kanupka JW, DeLong KS, Noel K. The impact of aquatic exercise on sleep behaviors in children with autism spectrum disorder: A pilot study. Focus Autism Other Dev Disabil. 2016;31(4):254–61.

[81] AdibSaber F, Ansari S. Comparing the Influence of an Aqua-based Versus a Mindfulness-based Kata Techniques Training on Sleep Habits and Stereotypic Behaviors in Children With Autism. 2024.

[82] Garcia JM, Murray M, Brazendale K, Rice DJ, Fukuda D. Effects of a family judo program on sleep quality in youth with Autism Spectrum Disorder. Sleep Med. 2024;115:152–4. 38367356 10.1016/j.sleep.2023.11.031

[83] NaraSiNgharao K, PradhaN B, NavaNeetham J. Efficacy of structured yoga intervention for sleep, gastrointestinal and behaviour problems of ASD children: An exploratory study. J Clin Diagn research: JCDR. 2017;11(3):VC01. 10.7860/JCDR/2017/25894.9502 PMC5427410 28511484

[84] Richter M, Taylor MK, Åkerlund S, Backman S, Krili S, Johansson BA, et al. Improved executive function and sleep quality in preteens with high-functioning autism following a structured physical activity program. Front Psychiatry. 2026;16–2025. 10.3389/fpsyt.2025.1726809. 10.3389/fpsyt.2025.1726809 PMC13033741 41918789

[85] Gudek Seferoglu E, Gurol A. Sleep Habits and Quality of Life of Intellectually Disabled Children with and without Regular Physical Activity. Bezmialem Sci. 2022;10(6):674–82. 10.14235/bas.galenos.2021.6090.

[86] Liu HLV, Sun F, Tse CYA. Examining the impact of physical activity on sleep quality in children with ADHD. J Atten Disord. 2023;27(10):1099–106. 37248735 10.1177/10870547231171723

[87] Li L, Wang C, Wang D, Li H, Zhang S, He Y, et al. Optimal exercise dose and type for improving sleep quality: A systematic review and network meta-analysis of RCTs. Front Psychol. 2024;15:1466277. 39421847 10.3389/fpsyg.2024.1466277 PMC11484100

[88] Toscano CV, Ferreira JP, Quinaud RT, Silva KM, Carvalho HM, Gaspar JM. Exercise improves the social and behavioral skills of children and adolescent with autism spectrum disorders. Front Psychiatry. 2022;13:1027799. 36620673 10.3389/fpsyt.2022.1027799 PMC9813515

[89] Tse AC, Lee PH, Zhang J, Chan RC, Ho AW, Lai EW. Effects of exercise on sleep, melatonin level, and behavioral functioning in children with autism. Autism. 2022;26(7):1712–22. 10.1177/13623613211062952. 35083939 10.1177/13623613211062952

[90] Brand S, Jossen S, Holsboer-Trachsler E, Pühse U, Gerber M. Impact of aerobic exercise on sleep and motor skills in children with autism spectrum disorders–a pilot study. Neuropsychiatr Dis Treat. 2015;1911–20. 10.2147/NDT.S85650 PMC4531010 26346856

[91] Wang T, Li W, Deng J, Zhang Q, Liu Y, Zheng H. The impact of the physical activity intervention on sleep in children and adolescents with neurodevelopmental disorders: a systematic review and meta-analysis. Front Neurol. 2024;15:1438786. 39193141 10.3389/fneur.2024.1438786 PMC11347421

[92] Tse ACY, Lee PH, Sit CHP, Poon ET-c, Sun F, Pang C-L, et al. Comparing the Effectiveness of Physical Exercise Intervention and Melatonin Supplement in Improving Sleep Quality in Children with ASD. J Autism Dev Disord. 2024;54(12):4456–64. 10.1007/s10803-023-06172-7. 37950776 10.1007/s10803-023-06172-7

[93] Owens J, Spirito A, McGuinn M. The Children’s Sleep Habits Questionnaire (CSHQ): psychometric properties of a survey instrument for school-aged children. Sleep. 2000;23(8):1043–51. 11145319

[94] Bruni O, Ottaviano S, Guidetti V, Romoli M, Innocenzi M, Cortesi F, et al. The Sleep Disturbance Scale for Children (SDSC). Construction and validation of an instrument to evaluate sleep disturbances in childhood and adolescence. J Sleep Res. 1996;5(4):251–61. 10.1111/j.1365-2869.1996.00251.x. 9065877 10.1111/j.1365-2869.1996.00251.x

[95] Forrest CB, Meltzer LJ, Marcus CL, de la Motte A, Kratchman A, Buysse DJ, et al. Development and validation of the PROMIS Pediatric Sleep Disturbance and Sleep-Related Impairment item banks. Sleep. 2018;41(6). 10.1093/sleep/zsy054. 10.1093/sleep/zsy054 29546286

[96] Phillips NL, Moore T, Teng A, Brookes N, Palermo TM, Lah S. Behavioral interventions for sleep disturbances in children with neurological and neurodevelopmental disorders: a systematic review and meta-analysis of randomized controlled trials. Sleep. 2020. 10.1093/sleep/zsaa040 32163581

[97] Meltzer LJ, Walsh CM, Peightal AA. Comparison of actigraphy immobility rules with polysomnographic sleep onset latency in children and adolescents. Sleep Breath. 2015;19:1415–23. 25687438 10.1007/s11325-015-1138-6 PMC4785891

[98] Kocanaogullari D, Akcakaya M, Bendixen R, Soehner AM, Hartman AG. What goes on when the lights go off? Using machine learning techniques to characterize a child’s settling down period. Front Netw Physiol. 2025;5. 10.3389/fnetp.2025.1519407. 10.3389/fnetp.2025.1519407 PMC12162617 40519639

[99] Goodman A, Goodman R. Strengths and difficulties questionnaire as a dimensional measure of child mental health. J Am Acad Child Adolesc Psychiatry. 2009;48(4):400–3. 19242383 10.1097/CHI.0b013e3181985068

[100] Hunter JE, McLay LK, France KG, Blampied NM. Systematic review of the collateral effects of behavioral sleep interventions in children and adolescents with autism spectrum disorder. Res Autism Spectr Disorders. 2020;79:101677. 10.1016/j.rasd.2020.101677.

[101] Woodford EC, France KG, Blampied NM, Hanning U, Swan CE, McLay LK. Behavioral sleep interventions for children with rare genetic neurodevelopmental conditions: A retrospective analysis of overall outcomes for 26 cases. Adv Neurodevelopmental Disorders. 2024;1–16.

[102] Duan Z, Wang X, Zhang Z, Wang X, Zhang Y, Du X. Digital and telehealth behavioral sleep interventions for improving sleep outcomes in children and adolescents with autism spectrum disorder: a systematic review and meta-analysis. Sleep Med. 2025;106870. 10.1016/j.sleep.2025.106870 41110404

[103] Corkum P, Lingley-Pottie P, Davidson F, McGrath P, Chambers CT, Mullane J, et al. Better nights/better days—distance intervention for insomnia in school-aged children with/without ADHD: a randomized controlled trial. J Pediatr Psychol. 2016;41(6):701–13. 27189687 10.1093/jpepsy/jsw031

[104] Ilie A, Orr M, Weiss S, Smith IM, Reid GJ, Hanlon-Dearman A, et al. Optimizing the Better Nights, Better Days for Children with Neurodevelopmental Disorders program for large scale implementation. Front Sleep. 2023;2:1158983. 41426450 10.3389/frsle.2023.1158983 PMC12713869

[105] Harvey AG. A Transdiagnostic Intervention for Youth Sleep and Circadian Problems. Cogn Behav Pract. 2016;23(3):341–55. 10.1016/j.cbpra.2015.06.001.

[106] Harvey AG, Buysse DJ. Treating sleep problems: A transdiagnostic approach. Guilford. 2017.

[107] Becker SP, Duraccio KM, Sidol CA, Fershtman CEM, Byars KC, Harvey AG. Impact of a Behavioral Sleep Intervention in Adolescents With ADHD: Feasibility, Acceptability, and Preliminary Effectiveness From a Pilot Open Trial. J Atten Disord. 2022;26(7):1051–66. 10.1177/10870547211056965. 34738484 10.1177/10870547211056965

[108] Kragh M, Dyrberg H, Kristiansen ST, Speed M, Pedersen P, Martiny K. Efficacy of a Transdiagnostic Sleep and Circadian Intervention for Outpatients With Sleep Problems and Depression, Attention Deficit Disorder, or Bipolar Disorder: A Randomised Controlled Trial. J Sleep Res. 2025;e70088. 10.1111/jsr.70088 PMC12856134 40345174

[109] Lenker KP, Harvey AG, Richdale AL, Calo WA, Fernandez-Mendoza J. Optimizing a Transdiagnostic Sleep and Circadian Intervention for Autistic Young Adults: A Qualitative Study Using an Implementation Science Framework. Behav sleep Med. 2026;1–17. 10.1080/15402002.2026.2658806. 10.1080/15402002.2026.2658806 41999360

[110] Roberts S, Ulmer CS. Barriers in Access to and Delivery of Behavioral Sleep Treatments. Curr Sleep Med Rep. 2024;10(1):70–80. 10.1007/s40675-023-00270-9.

[111] Yoo I. A scoping review of sleep management as an occupational therapy intervention: expanding a niche area of practice in mental health. Ir J Occup Therapy. 2023;51(2):22–34. 10.1108/IJOT-01-2023-0001.

[112] McLennan H, Aberdein R, Saggers B, Gillett-Swan J, Neurodiversity. A scoping review of empirical research. Neurodiversity. 2025;3:27546330251337874.

[113] Malkani MK, Sheridan AM, Crichton AJ, Bucks RS, Pestell CF. In-person versus online delivery of a behavioral sleep intervention (Sleeping Sound©) for children with ADHD: protocol for a parallel-group, non-inferiority, randomized controlled trial. BMC Pediatr. 2023;23(1):502. 37803298 10.1186/s12887-023-04329-y PMC10557213

[114] Davis NO, Eichner B, Gibson MJ, Lunsford-Avery JR. Preschool attention and sleep support (PASS): protocol for a pilot feasibility randomized clinical trial. Front Sleep. 2025;4:1662221. 10.3389/frsle.2025.1662221. 41728023 10.3389/frsle.2025.1662221 PMC12920245

[115] Joseph HM, Levenson JC, Conlon RPK, Mannion K, Kipp HL, Gradian A, et al. Optimizing Attention and Sleep Intervention Study (OASIS): a protocol for a pilot randomized controlled trial to compare parent behavioral interventions with and without sleep strategies delivered in pediatric primary care for preschool-aged children at risk of childhood ADHD. Pilot Feasibility Stud. 2025;11(1):22. 10.1186/s40814-025-01600-0. 40001155 10.1186/s40814-025-01600-0 PMC11852881

[116] Keuppens L, Marten F, Baeyens D, Boyer B, Danckaerts M, van der Oord S. Sleep IntervEntion as Symptom Treatment for ADHD (SIESTA)-Blended CBT sleep intervention to improve sleep, ADHD symptoms and related problems in adolescents with ADHD: Protocol for a randomised controlled trial. BMJ Open. 2023;13(4):e065355. 37055205 10.1136/bmjopen-2022-065355 PMC10106018

